# Effects of Peptidase Treatment on Properties of Yeast Protein as an Alternative Protein Source

**DOI:** 10.4014/jmb.2409.09062

**Published:** 2024-11-18

**Authors:** Ju Hyun Min, Nur Istianah, Jeong Hwa Jang, Hyeon Ji Jeon, Young Hoon Jung

**Affiliations:** 1 School of Food Science and Biotechnology, Kyungpook National University, Daegu 41566, Republic of Korea; 2 Department of Food Science and Biotechnology, Brawijaya University, Malang 65145, Indonesia

**Keywords:** Yeast protein hydrolysates, endotype protease, exotype protease, hydrolysis, alternative protein

## Abstract

Yeast protein, high-quality and high-content microbial protein, can serve as alternative sources of protein. This study examined the structural and functional characteristics of yeast protein through enzymatic treatment using different ratios of alcalase (endo-type) and prozyme 2000P (exo-type) including 2:1 (A2P1), 1:1 (A1P1), and 1:2 (A1P2). After enzymatic hydrolysis, a significant increase in protein solubility from less than 3.1% in untreated proteins to around 16%, particularly at pH 2 or pH 12. Furthermore, a maximum degree of hydrolysis of over 85% was achieved after enzyme treatment. Among them, the highest value of 87.73% was achieved at yeast protein treated by A1P2. Scanning electron microscopy images revealed varied surface morphologies, with exhibiting an increased surface area, particularly after treatment using A2P1. Next, yeast protein treated with A2P1 also demonstrated a superior emulsion stability index (3364.17). However, the antioxidant capacity was higher in proteins treated with A1P2 (78.30%). In addition, the elevated levels of certain amino acids, specifically leucine, lysine, phenylalanine, valine, and arginine, thereby indicating an enhanced amino acid profile was observed. Overall, yeast proteins treated with complex enzymes exhibited improved functionality and potential for diverse food applications.

## Introduction

As awareness of the nutritional importance of protein increases, there is an escalating demand for protein sources, encompassing both plant-based and animal-derived options [1]. However, the widespread production of high-quality animal protein globally poses significant environmental sustainability concerns [2, 3]. Consequently, there is a mounting emphasis on incorporating more alternative proteins into diets to provide sufficient high-quality protein; while, potentially mitigating environmental impacts [3]. As a type of microbial proteins, yeast protein presents a promising alternative owing to its rapid growth, high protein content, minimal risk of contamination, and ease of harvest, attributed to its small cell size and cohesive properties [4, 5]. Although there have been extensive researches on utilizing yeast protein in animal feed, exploring the potential applications of protein hydrolysates remains relatively limited [6-8].

Enzyme reactions have remarkable advantages such as high catalytic activity and precise substrate specificity, rendering them ideal biocatalysts for decomposing bioactive peptides in food and accelerating the reaction rate by approximately 10^6^ to 10^12^ times compared to uncatalyzed reactions [9]. Biological methods are favored over chemical agents because of the non-corrosive, non-flammable, and non-toxic nature, which ensures safety for both the environment and human health [10, 11]. In contrast to chemical hydrolysis, which results in nonspecific breakdown into peptides and amino acids (AAs), the enzymatic approach enables a highly precise and controlled cleavage of specific amide bonds [12]. Ultimately, utilizing biological processes for protein breakdown offers a preferred method for producing a variety of small and large peptides in the diets of livestock, poultry, and fish. This approach not only provides nutritional benefits but also fulfills essential physiological and regulatory functions [13, 14].

Proteolytic enzymes can be divided into two main types based on their positional specificity: endopeptidases and exopeptidases. Endopeptidases break peptide bonds within the interior of polypeptide chains, while exopeptidases act on the terminal regions, cleaving bonds close to either the C-terminus or N-terminus [15]. Previous study showed that among different types of enzymes such as endo- (alcalase and neutrase) and exotype (flavourzyme and prozyme 2000P), alcalase was producing smaller particle sizes and higher levels of amino acids as well as prozyme 2000P showed faster hydrolysis over time, higher solubility, better hydrolysis efficiency, and even smaller particle sizes [15]. Similarly, Dryáková *et al*. (2010) demonstrated that for whey protein hydrolysates, alcalase exhibited a higher degree of hydrolysis and a lower average chain length than neutrase among endo-type enzymes [16]. Furthermore, Kim *et al*. (2021) found that for chicken breast hydrolysates, prozyme 2000P achieved higher hydrolysis efficiency than flavourzyme [17].

In general, researches have been focused using a single protein-degrading enzyme for substrate hydrolysis. However, recent studies indicate a growing interest in exploring the combined use of enzymes (*e.g.* endo-type and exo-type) [18-22]. This is because the cleavage of peptide bonds by endopeptidases increases the number of terminal peptide regions available for exopeptidase activity [23]. Furthermore, studies have reported that the simultaneous treatment of alcalase and prozyme for the hydrolysis of whey protein produces more amino nitrogen compared to using them individually, thereby improving digestion efficiency [24]. Nevertheless, there remains a need to compare the effects of hydrolysis using different groups of enzymes on the structural and functional characteristics of yeast protein, especially in terms of alternative protein sources.

Therefore, this study aimed to evaluate the effects of enzyme treatments, either individually or in combination (endo- and exo-types), on the properties of yeast proteins during the production of enzymatic protein hydrolysates as a protein source. This will provide fundamental data for exploring their potential application as an alternative to animal protein.

## Materials and Methods

### Materials

Yeast protein was generously provided by Amored Fresh (Republic of Korea). Proteolytic enzymes, including the alcalase 2.4 L FG from *Bacillus licheniformis* (endopeptidase) and prozyme 2000P from plants (exopeptidase) were purchased from Bision Biochem Corporation (Republic of Korea). Bromophenol blue was obtained from Duksan Chemicals (Republic of Korea), trichloroacetic acid from TCIchemical (Republic of Korea), and the bicinchoninic acid (BCA) protein assay kit from Thermo Fisher Scientific (Republic of Korea). Soybean oil was purchased from Ottogi Co. Ltd., Republic of Korea.

### Preparation of Yeast Protein Hydrolysate through Proteolysis

To measure the proteolysis effect of the yeast protein, enzyme mixtures were added to 10% (w/v) yeast protein solution at an enzyme:substrate ratio of 1:100; then, incubated at 55°C since the optimal reaction temperatures for alcalase and prozyme 2000P are 55–70°C and 55–60°C, respectively. Specifically, alcalase was applied for the initial 6 h, after which prozyme 2000P was added for an additional 4 h. Thus, the total reaction time was 10 h, with each enzyme applied sequentially for its designated duration (6 h for alcalase and 4 h for prozyme 2000P). This setup was based on preliminary studies monitoring pH changes, which helped determine the optimal treatment times for each enzyme. The enzyme mixtures contained both alcalase and prozyme 2000P at different ratios of 1:1, 1:2, and 2:1, respectively. After the hydrolysis reaction, the protein hydrolysate was frozen at -40°C and subjected to freeze-drying for 3 d using a freeze dryer, following a modified analytical method. All samples were stored at room temperature for further analysis [18].

### Degree of Hydrolysis (DH)

To determine the DH after enzyme reaction, a 1% (w/v) hydrolysate solution adjusted to pH 7 using NaOH was incubated at room temperature for 30 min after the addition of an equal volume of 20% (w/v) trichloroacetic acid solution, and then centrifuged (Eppendorf 5910 R, Germany) at 2,634 ×*g* and 4°C for 20 min. The protein concentrations in the supernatant were measured at 562 nm using an ultraviolet-visible spectrophotometer (Epoch 2 Microplate Spectrophotometer, BioTek, USA), according to the BCA method (Smith *et al*., 1985). The DH value was calculated based on the following Eq. (1) [25]:

$$\text{DH}\left(\%\right)=\frac{\left(OD_{h}-OD_{0}\right)}{OD_{h}}\times 100$$ (1)

where OD_o_ and OD_h_ denotes the absorbance of the yeast protein before and after hydrolysis, respectively.

### Determination of Solubility

The solubility of the hydrolysate obtained after enzyme treatment were investigated [26, 27]. A 1% (w/v) protein solution was incubated for 30 min at room temperature across a pH range from 2 to 12, which was achieved through adjustments with 1 M HCl and 1 M NaOH solutions. Following incubation, the solution was centrifuged at 2,634 g for 25 min, and then, the protein content in the resultant supernatant was quantified using the protein quantitation kit (Pierce BCA Protein Assay Kit, Thermo Fisher Scientific, USA). Protein solubility was calculated as the percentage of soluble protein concentration relative to the total protein concentration in the sample.

### Particle size Distribution (PSD)

PSD analysis was performed using a particle size analyzer (Mastersizer 3000, Malvern Panalytical Ltd., UK), covering a size range of 0.01–3500 μm. A red laser (633 nm) and a blue light source (470 nm) were utilized for measurements. PSD results, including the D_10_, D_50_, and D_90_ values, where D_10_, D_50_, and D_90_ correspond to the 10th, 50th, and 90th percentiles, respectively [28, 29], were determined using Mastersizer 3000 software and are presented in a volume-based format. The distribution width, indicated by the span, was calculated as (D_90_–D_10_)/D_50_.

### Size exclusion Chromatography

The average molecular weights (M_w_ and M_n_) of yeast protein were measured using size exclusion chromatography (Alliance e2695; Waters, USA), with a column (Ultrahydrogel column, Waters, USA) and a refractive index detector (2414 RI detector, Waters). A mobile phase of 0.02 N NaNO_3_ was flowed at 0.8 ml/min. M_w_ was determined using a calibration curve based on standard pullulan samples (63-642 kDa).

Two distinct methods exist for calculating molecular weight, with the most significant being the number-average molecular weight, Mn. For any given molecules, i, within a sample, the number-average molecular weight is determined through Eq. (2).

$$M_{n}=\frac{\sum_{i}N_{i}M_{i}}{\sum_{i}N_{i}}$$ (2)

In this context, i signifies the count of polymer molecules, and N_i_ indicates the number of molecules that possess the molecular weight M_i_.

Conversely, the calculation for weight-average molecular weight differs, as illustrated in Eq. (3) [30].

$$M_{w}=\frac{\sum_{i}N_{i}M_{i}^{2}}{\sum_{i}N_{i}M_{i}}$$ (3)

### UV Spectra Analysis

The UV spectra of the yeast protein hydrolysates were examined to identify structural changes after enzymatic treatment [31]. Yeast protein suspensions, at a concentration of 2 g/l prepared in 0.05 mol/l Tris-HCl buffer at pH 8.0, were centrifuged at 3,900 ×*g* for 20 min. The supernatant was then scanned using a UV–Vis spectrophotometer (Epoch2 Microplate Spectrophotometer, BioTek, USA) across a wavelength range of 200–400 nm at a scanning rate of 1 nm/min.

### SEM and FTIR

Morphological properties were assessed using field emission-scanning electron microscopy (SEM; Hitachi SU8030, Hitachi, Japan). Samples were prepared by placing them on aluminum foil, drying at room temperature for 12 h, and coating with osmium before imaging at an accelerating voltage of 5 kV [32].

Fourier transform-infrared (FTIR; Nicolet iS5 FTIR Spectrometer, Thermo Scientific, USA) spectroscopy was used to obtain spectra in transmission mode within the range of 4,000 cm^-1^ to 550 cm^-1^ with a resolution of 4 cm^-1^ and averaging over 16 scans [33].

### Analysis of the Degree of Molecular Weight Distribution through Protein Degradation

The molecular weight distribution of yeast protein after enzymatic hydrolysis was determined using modified sodium dodecyl sulfate-polyacrylamide gel electrophoresis (SDS-PAGE) [34]. The protein samples were dissolved in 5% SDS and incubated for 1 h. The solubilized proteins were then mixed in a 1:1 v/v ratio with 0.5 M Tris-HCl, pH 6.8, containing 20% glycerol and 5% SDS sample buffer. The SDS-PAGE was prepared with an 8% or 14% separating gel and a 4% stacking gel. After loading 15 μl of samples on a polyacrylamide gel, electrophoresis was performed at 120 V and/or 150 V. Protein bands were stained using Coomassie brilliant blue R-250 (Bio-Rad Laboratories, Inc., Republic of Korea) and destaining was performed using 50% v/v methanol and 7.5% v/v acetic acid to visualize the protein bands. Precision plus protein dual xtra standards (Bio-Rad Laboratories, Inc.) were used as a control.

### ABTS Radical Scavenging Activity

The antioxidant capacity of the treated construct was assessed using the ABTS radical scavenging assay. To generate 2,2'-azinobis(3-ethylbenzothiazoline-6-sulfonate) radical anion (ABTS•^+^), a mixture of ABTS stock solution (7.4 mM) and potassium persulfate (2.6 mM) in phosphate-buffered saline (PBS, pH 7.4) was prepared and incubated in darkness at room temperature for 18 h. A 100 μl aliquot of the extracted sample was mixed with 100 μl of ABTS solution in a 96-well plate, and the absorbance was measured at 734 nm. The scavenging activity (%) was calculated using the following Eq. (4) [35].

$$\text{Scavenging activity (\%)}=\frac{\left(A_{control}-A_{sample}\right)}{A_{control}}\times 100$$ (4)

where A_sample_ is the absorbance of the ABTS solution with the sample, and A_control_ is the absorbance of the ABTS solution with 95% ethanol.

### Water Holding Capacity (WHC) and Oil binding Capacity (OBC)

The WHC and OBC of the protein hydrolysates were assessed using a method previously documented [36, 37]. For WHC, dispersions of 0.02 g/g were prepared using the protein concentrates; while for OBC, dispersions of 0.2 g/ml were prepared. These dispersions were shaken overnight at room temperature and then centrifuged at 2,058 ×rcf for 30 min at 20°C. The supernatant was removed, and the weight of the wet pellet was noted. Subsequently, after freeze-drying, the weight of the dry pellet was also recorded. The WHC_insoluble_ and OBC were determined according to the Eqs. (5) and (6), respectively:

$$WHC_{inso\mathrm{lub}le}\left(g/g\right)=\frac{\left(m_{wet\;pellet}-m_{dry\;pellet}\right)}{m_{dry\;pellet}}$$ (5)

$$OBC\left(g/g\right)=\frac{\left(W_{f}-W_{i}\right)}{W_{i}}$$ (6)

where m_wet pellet_ is the mass of the pellet after centrifugation and before drying, m_dry pellet_ is the mass of the pellet after drying, the W_f_ and W_i_ are the final weight of the pellet after centrifugation and the initial weight of the dry powder, respectively.

### Emulsifying Properties of Yeast Protein Treated with Single or Complex Protease

The emulsifying activity index (EAI) and emulsion stability index (ESI) of yeast protein were assessed according to a modified method [38]. In brief, 12 ml of yeast protein solution was mixed with 4 ml of soybean oil to achieve an oil fraction of 0.25. This mixture was homogenized using an ultrasonicator (KSS-750DT, korprotec, Republic of Korea) at 20 kHz for 2 min. Subsequently, 50 ml of the freshly prepared emulsion was extracted from the bottom and diluted with 5 ml of 0.1% SDS. After thorough vortexing, the absorbance was measured at 500 nm. The EAI and ESI were calculated using Eqs. (7) and (8):

$$EAI(m^{2}/g)=\frac{2\times 2.303\times A_{0}}{0.25\times protein\;weight(g)}$$ (7)

$$ESI(\min)=\frac{A_{0}}{A_{0}-A_{30}}\times \Delta T$$ (8)

where A_0_ and A_10_ represent the absorbance of the diluted sample at 0 and 30 min, respectively, and ΔT equals 30 min.

### Determination of SH Group Levels

In determining the SH group content, two sets of Tris buffers were prepared. The first set (buffer 1) included 10.4 g of Tris, 6.9 g of glycine, and 1.2 g of ethylene-diamine-tetraacetic acid (EDTA) dissolved in one liter of deionized water, adjusted to pH 8.0. The second set (buffer 2) was identical to the first but included 480 g of urea. Ellman’s reagent was prepared by mixing 0.2 g of 2-nitrobenzoic acid (DTNB) with 50 ml of the buffer 1. To analyze the content of sulfhydryl groups on surface and in free form, the precipitate was dissolved in 5 ml of buffer 1 and buffer 2, respectively. A mixture consisting of 0.1 g of yeast protein powder, 2.0 ml of either buffer 1 or 2 and 0.02 ml of Ellman’s reagent was allowed to react at 25°C for 5 min. The absorbance at 412 nm was measured using a UV–Vis spectrophotometer. For the baseline, solutions without yeast protein served as blanks; while, solutions with yeast protein but without Ellman’s reagent were used to assess turbidity. The SH group level was expressed as follows:

$$\text{SH group level }(\mu mol/g)=(75.53\times A_{412}\times D)/C$$ (9)

where A_412_ denotes the difference in absorbance at 412 nm with and without DTNB in the color-developing solutions. D denotes the dilution factor (for SH, D=3.02), and C denotes the total solids content of yeast protein [39].

### Composition of Free Amino Acids (AA)

The composition of free AAs within the yeast-protein extract was analyzed using a high-performance liquid chromatography system (Dionex Ultimate 3000, Thermo Fisher Scientific), coupled with a 1260 Infinity fluorescence detector (Agilent Technologies). After derivatization using o-phthalaldehyde (OPA) and 9-fluorenylmethoxycarbonyl (FMOC) [40, 41], samples were injected into an Inno-C18 column and detected at specific excitation (340 nm) and emission wavelengths (450 nm) for OPA and FMOC derivatives. A gradient program employing a mobile phase of 40 mM sodium phosphate (pH 7) and a mixture of water, acetonitrile, and methanol was used [42].

### Statistical Analysis

Statistical analysis was performed using MINITAB version 21. All measured parameters were subjected to a one-way analysis of variance, followed by Tukey’s post-hoc test to identify significant differences among the mean values of each group. Statistical significance was established at a level of *p* < 0.05.

## Results and Discussion

### Analysis of Yeast Protein Solubility and Degree of Hydrolysis (DH)

**Fig. 1:**
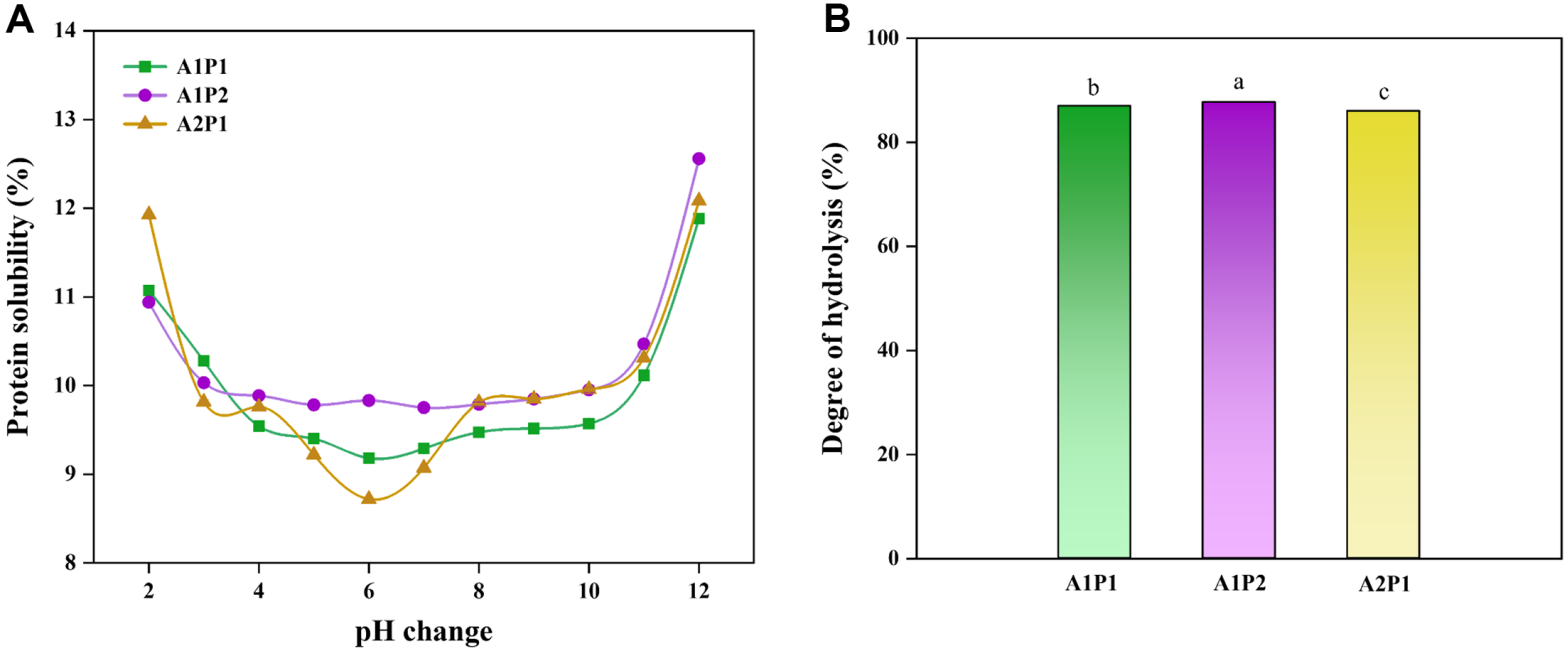
Enzyme hydrolysis of yeast protein. (**A**) protein solubility, (**B**) degree of hydrolysis. Different letters indicate statistically significant differences (*p* ≤ 0.05).

The solubility of proteins plays a crucial role in various functional properties of proteins, such as their emulsification and foaming capabilities, by influencing their ability to migrate to oil-water or air-water interfaces [43], which are essential for maintaining product stability and quality throughout processing, storage, and consumption, as well as which are not readily achievable with insoluble proteins. In the present study, the protein solubility was measured at different pH levels at 55°C for a total of 10 h reaction (6 h for prozyme 2000P and then additional 4 h for alcalase with prozyme 2000P) based on a previous study [15]. During the reaction, the release of amino acids results in a gradual decrease in pH and then was stabilized, after a certain period such as approximately 4 h for Alcalase and 6 h for Prozyme 2000P. The solubility of yeast proteins hydrolyzed by different combinations of alcalase and prozyme 2000P at 2:1 (A2P1), 1:1 (A1P1), 1:2 (A1P2) at different pH levels has shown that the solubility of untreated yeast proteins was limited to a range of 0.9% to 3.1% [15]; while, enzymatically treated yeast proteins showed about 9% to 16% solubility, which is equivalent to an about 10-fold increase in solubility (Fig. 1A). Notably, the highest solubility was observed at a pH of 2 with A2P1 and at a pH of 12 with A1P2. Around the isoelectric point (pH 4–6), at which a protein, peptide, or any amphoteric solute has a net neutral charge, showed minimal solubility. At this point, the weakening of the electrical double layer diminishes electrostatic repulsion between protein molecules, which, in low ionic strength solutions, may result in protein precipitation [44]. Conversely, plant proteins typically show increased solubility as the pH moves away from their isoelectric point due to the altered charge inducing greater electrostatic repulsion and thus showing relatively higher solubility [43, 45]. Therefore, the high solubility of the yeast protein achieved through this enzyme treatment can enhance the utilizability in various food industries including beverages, dairy products, sauces, and processed meat products.

The degree of hydrolysis (DH) is essential for determining the proportion of peptide bonds cleaved within a protein hydrolysate, serving as a critical parameter for identifying structural differences among various hydrolysates [46]. Overall, the DH values of the yeast proteins treated with complex enzymes (alcalase and prozyme 2000P) showed over 85% [15]. The enzymatically treated yeast proteins at A1P2 exhibited the highest DH value of 87.73% (Fig. 1B). In previous studies, whey protein hydrolysates treated with alcalase and prozyme 2000P achieved a 50% degree of hydrolysis after 8 h, demonstrating the high efficiency of using combined enzyme for the production of yeast protein hydrolysates [28].

### Gel Electrophoretic Examination of Yeast Protein Hydrolysate Profiles

**Fig. 2:**
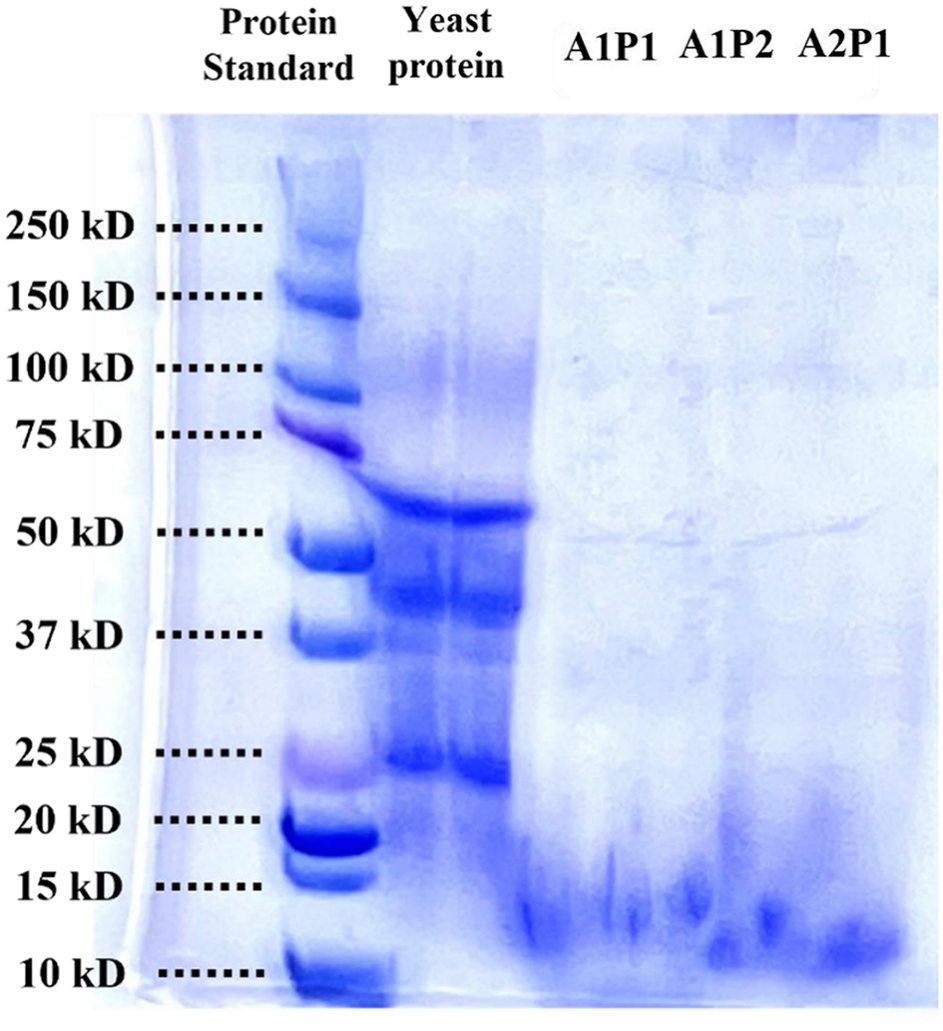
SDS-PAGE patterns with enzyme hydrolysis of yeast protein.

SDS-PAGE was utilized to investigate the hydrolytic properties of yeast proteins. Protein bands within the range of 10–250 kD were observed (Fig. 2). For untreated yeast protein, distinct bands were observed around 25, 40, and 60 kD. After enzyme treatment, A2P1 showed bands across the 15–25 kD range; while, A1P2 exhibited bands across the 15–20 kD range, which is in good agreement with hydrolysis results. This difference is attributed to the hydrolysis mechanisms of the enzymes; endopeptidases hydrolyze internal peptide bonds, whereas exopeptidases target the N- or C-termini [47]. Alcalase likely produced larger fragments compared to prozyme 2000P in this study. These findings are consistent with previous studies on mealworm proteins, where samples treated with single and complex enzymes showed similar band positions, but samples treated with complex enzymes appeared fainter [48].

### Morphological Examination of Yeast Protein Hydrolysates

**Fig. 3:**
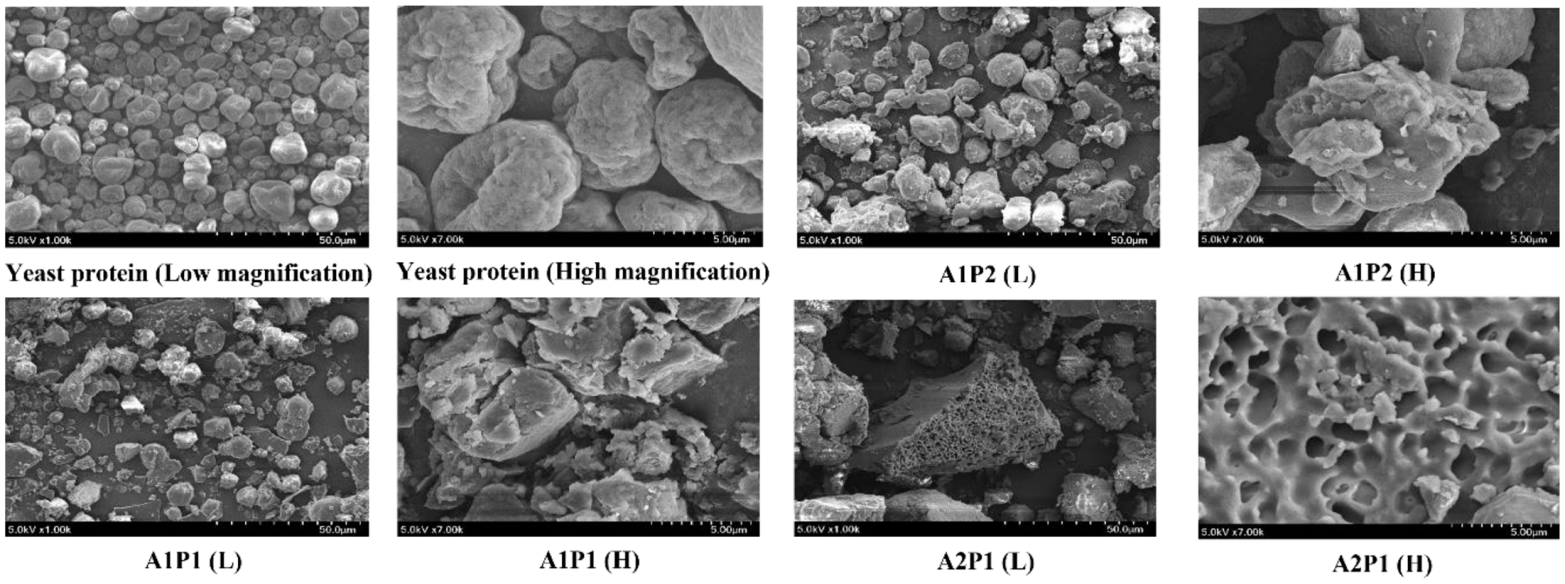
Scanning electron microscopic images of yeast protein treated with various ratio of enzyme.

The microstructure of yeast protein (Fig. 3) varied significantly after treatment with complex enzymes. Untreated yeast protein displayed smooth surfaces and spherical particles, particularly showing uniformity in size. However, enzymatically hydrolyzed yeast protein revealed small protein fragments with uneven sizes [49, 50]. Particularly, proteins treated with A2P1 showed larger fragments similar to the results in Fig. 2. Also, complex enzyme treatment using showed the emergence of porous surfaces in yeast protein, which might increase the surface area of protein hydrolysate, potentially enhancing solubility by providing more contact opportunities with water [51].

### Effects of Hydrolysis on the Particle Size and Molecular Weight

Table 1 presents the average particle size distribution of the yeast protein hydrolysate treated by complex enzymes. Compared to the particle sizes of untreated yeast protein (D_50_: 12.80 μm), the enzymatically treated yeast protein demonstrated smaller particle sizes such as D_50_ value of from 9.76 μm to 10.04 μm [15]. Similarly, gel permeation chromatography (GPC) results indicated that proteins treated with complex enzymes had higher M_w_ (weight-average molar mass) than untreated proteins (M_w_: 6446) (Table 2). Reducing particle size increases the surface area available for interaction with digestive enzymes, thus probably enhancing nutrient digestibility [52, 53]. Meanwhile, the span value which is an important measure for evaluating the differences between D_10_ and D_90_ values, reflected a more consistent particle distribution [54]. In the referenced study, samples treated with flavourzyme and prozyme 2000P (exo-type enzymes) had an average span value of 1.81; while, those treated with alcalase and neutrase (endo-type enzymes) had an average value of 1.68 [15]. This suggests that endo proteases might significantly affect particle size reduction in yeast protein treated with complex enzymes.

### Structural and Compositional Changes in Yeast Protein Hydrolysates

**Fig. 4:**
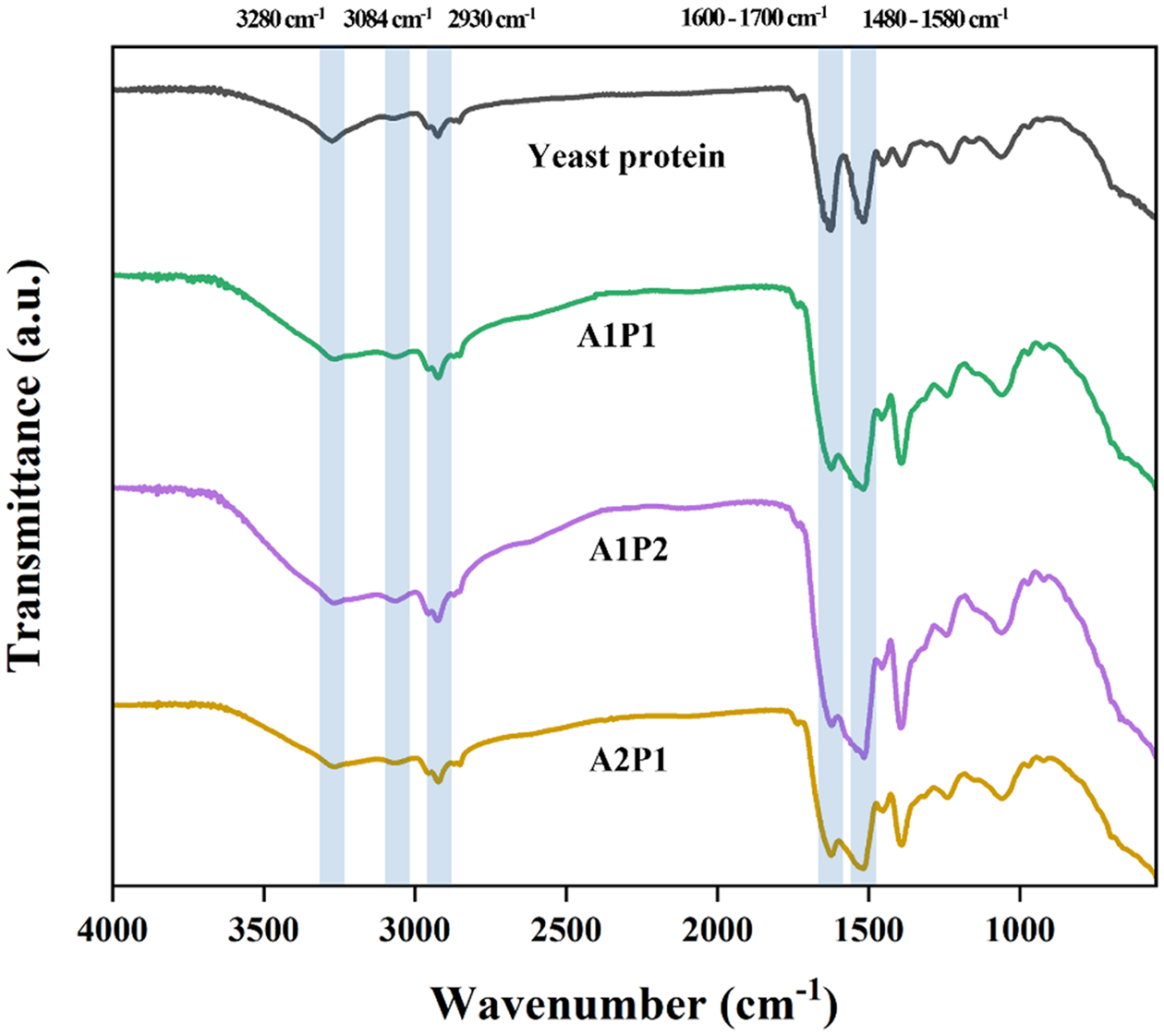
Fourier transform infrared analysis with yeast protein treated by enzyme hydrolysis.

The FTIR spectra presented in Fig. 4 investigated changes in characteristic peaks corresponding to Amide A, I, II, and III in yeast protein hydrolysates. Notably, untreated yeast protein exhibited a distinct peak at 3280 cm^−1^, 2930 cm^−1^, 1650 cm^−1^, and 1530 cm^−1^.

The peak aligning with the wavenumber range (3500–3200 cm^−1^) suggests the presence of N–H stretching vibration associated with amide A [55, 56]. The amide I peak, associated with C=O stretching vibration, is within the 1700–1600 cm^−1^ range. Amide II peak, corresponding to N–H bending and C–H stretching vibrations, appears within the 1580–1480 cm^−1^ range. The presence of amide I and amide II in yeast protein and its hydrolysate was confirmed by peaks at 1630 and 1520 cm^−1^, respectively. In addition, by reduction of the peaks at 1630 cm^−1^, the enzyme treatment make reduction in the protein’s hydrogen-bonding environments (α-helix, β-sheet, turn, and unordered conformations) [57]. Additionally, bands observed at 2930 cm^−1^ correspond to –CH_2_ groups [58].

The structural changes by enzyme treatment were also observed in reduction of sulfhydryl group contents (Table 3). The sulfhydryl (SH) group is a vital functional group in proteins, reflecting the degree of protein denaturation and significantly impacting protein functional properties [59]. Regardless of enzyme ratio, the levels of surface SH and total free SH groups in yeast proteins hydrolyzed by various enzyme treatments, implying disulfide bonds in protein folding and structures were reduced. Particularly, the proteins treated with A2P1 showed the lowest free SH group and the highest surface SH group, implying the highest protein denaturation but the highest reaction possibility like stabilizing gel structure [60]. Meanwhile, since the intensity of S–S stretching absorption is in general weak and significantly affected by the structural environment, it did not appear in the FTIR spectrum [61, 62].

**Fig. 5:**
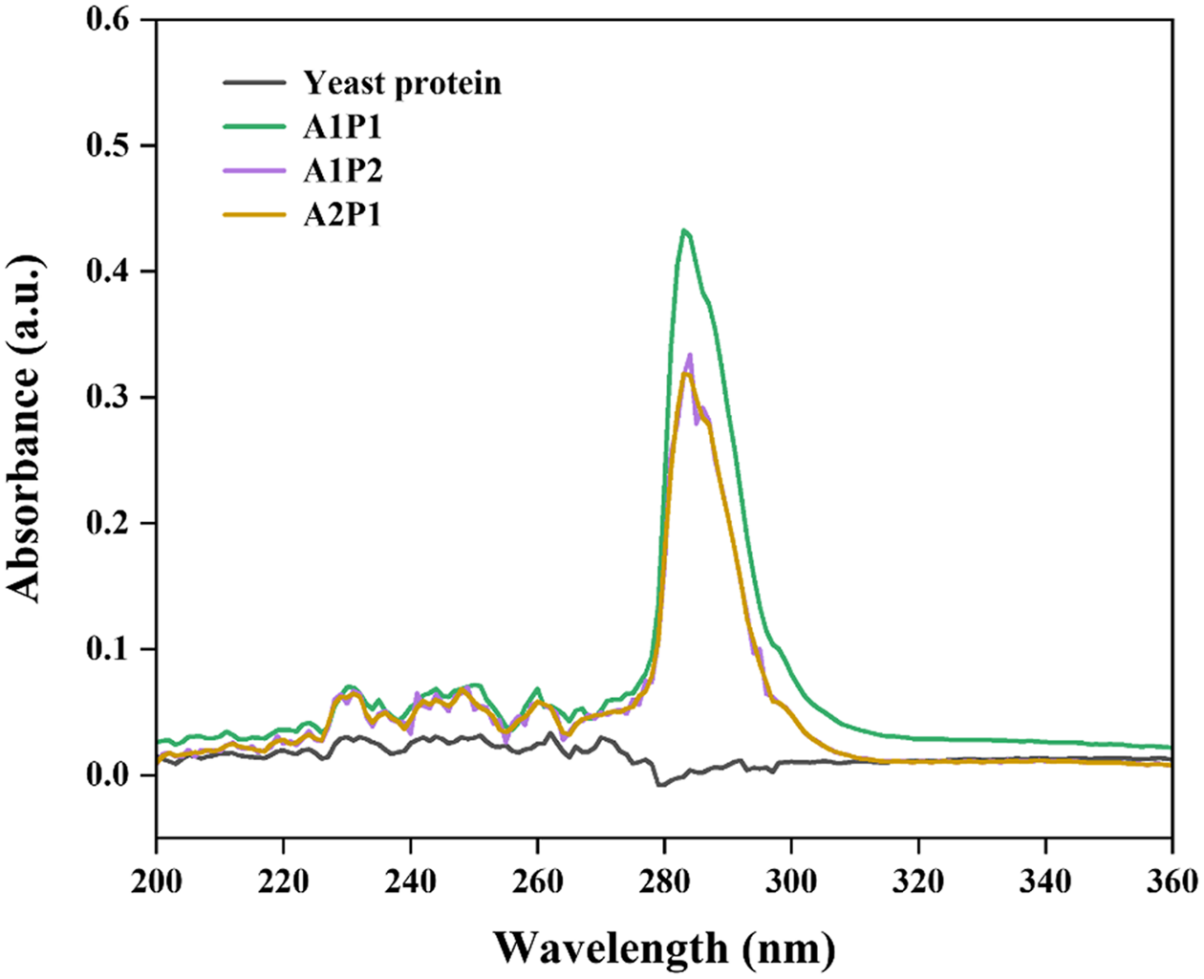
UV-spectra scanning of yeast protein treated with complex enzymes.

These structural changes were also observed by investigating UV absorption spectra of yeast protein hydrolysates (Fig. 5). Unlike untreated yeast protein showing no absorption peaks at between 200 and 360 nm, enzymatic hydrolysis caused notable changes in the UV absorption bands with yeast protein, in that, an absorbance of > 0.3 was detected at an indicative peak of 283 nm at all the complex enzyme treated protein hydrolysates. These changes are likely due to protein conformation alterations following enzyme treatment [63, 64], which are also in accordance with amino acid profiles (Table 4). The absorption of near UV radiation by proteins is primarily influenced by the presence of tyrosine (Tyr) and tryptophan (Trp) residues, with minor contributions from phenylalanine (Phe) and disulfide bonds, affecting the A_280_ value [65]. As an example, since Tyr, Trp, and Phe levels in pure yeast proteins had been reported to be almost undetectable (below 20.17 mg/kg)[15], the UV absorption spectra of untreated yeast proteins could not be examined.

Furthermore, beyond increase in hydrophobic amino acids content, total free amino acids content in enzymatically hydrolyzed yeast protein exhibited over 400 times higher value, in comparison with the amino acid levels in untreated yeast proteins ranged from 1.32 to 117.01 [15]. Accordingly, all the amino acids values including total essential amino acids displayed higher values regardless of enzyme complex introduced. Following the criteria set by the Food and Agriculture Organization and the World Health Organization, proteins treated with complex enzymes showed a high-quality profile, achieving or surpassing the reference value of essential amino acids/total amino acid ≥40% [66]. This indicates the effectiveness of complex enzyme treatment in improving protein quality over untreated yeast protein [15]. Notably, the amino acids in yeast proteins treated with complex enzymes, especially leucine, lysine, phenylalanine, valine, and arginine, were significantly higher. A previous study indicated that animal proteins typically have higher levels of lysine and valine than plant proteins, suggesting that yeast proteins treated with complex enzymes could potentially serve as viable alternatives to animal proteins [67].

### Functional Properties Yeast Protein Hydrolysates as an Alternative Source

**Fig. 6:**
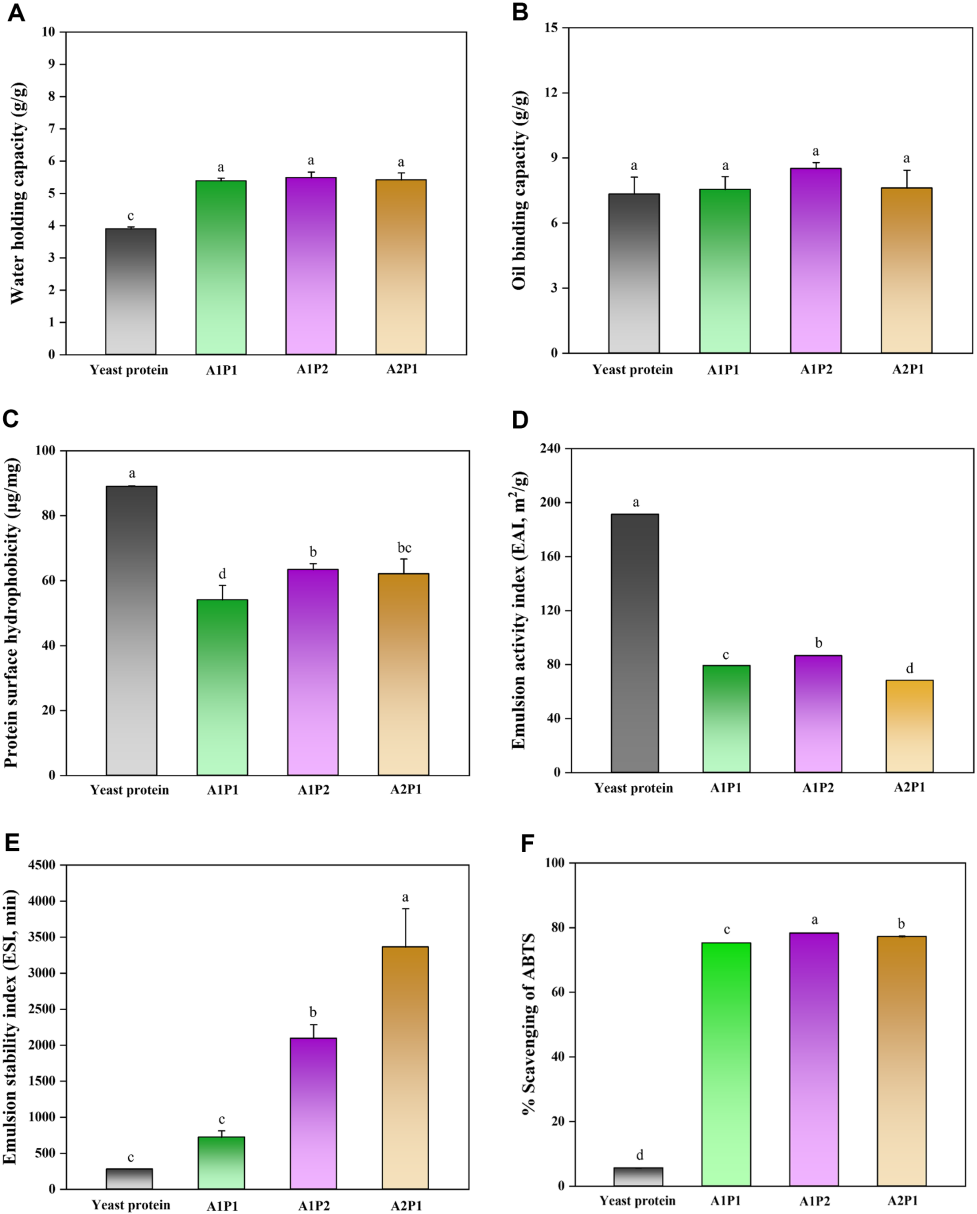
(A) Water holding capacity, (B) oil binding capacity, (C) protein surface hydrophobicity, (D) emulsifying activity index, (E) emulsion stability index, and (F) the capacity of radical scavenging on ABTS of yeast protein treated with complex enzyme. Different letters indicate statistically significant differences (*p* ≤ 0.05).

The effects of complex enzyme treatment on the different properties of yeast protein hydrolysates regarding surface reactivity and emulsifying ability were investigated (Fig. 6). However, untreated yeast proteins had the lowest WHC, yeast protein hydrolysates enzymatically treated showed increased values in WHC (Fig. 6A). The increase in WHC is probably because of the exposure of polar groups including -COOH and -NH_2_ during enzymatic hydrolysis [68]. In addition, the enhancement in OBC could be explained by alterations in the protein's secondary structure because of enzymatic hydrolysis. During this process, non-polar side chains may have become exposed, enabling them to bind with fat molecules. The increased surface area of the protein particles and the exposure of hydrophobic groups caused by the unfolding of protein chains are likely the key factors contributing to the improved oil binding capacity [69]. Protein powders with smaller sizes in general exhibit better water and oil adsorption and entrapment capabilities than those with high density. Therefore, significant differences were observed in OBC levels compared to untreated yeast proteins (Fig. 6B). Despite significant structural changes (or size reduction) due to protein hydrolysis, the surface hydrophobicity of enzyme-treated yeast protein decreased (Fig. 6C). Short-term hydrolysis can induce protein unfolding and increase hydrophobicity by exposing previously hidden hydrophobic groups. However, prolonged hydrolysis, such as the 10-hour treatment in this study, may lead to the breakdown of hydrophobic regions and the refolding of hydrophobic groups in cleaved peptide fragments, thereby reducing surface hydrophobicity [70]. Thus, the OBC increased due to the hydrophobic groups within the hydrolyzed protein molecules, but surface hydrophobicity decreased compared to untreated yeast protein.

Next, emulsifying ability of the protein hydrolysates after complex enzyme treatment was investigated (Fig. 6D-6E). The EAI is defined as the surface area of the stabilized oil/water interface per unit weight of protein; while, the ESI measures the resistance of the emulsion over time [71, 72]. In this study, untreated yeast proteins showed the highest EAI, but the lowest ESI. Among the enzyme-treated samples, the highest EAI was observed in A1P2. This might be because proteins with larger molecular weight forming a thicker coating around oil droplets, whereas excessive hydrolysis leads to weaker interactions among shorter peptides. These characteristics suggest that the enzyme-treated protein could help maintain product quality over an extended period in fermented foods or products requiring long shelf lives, such as dressings, sauces, and mayonnaise.

Furthermore, since highly hydrophilic peptides exhibit no affinity for the droplets and remain in the solution [73], the untreated yeast protein with the highest emulsifying activity showed the lowest ESI. Conversely, yeast proteins treated with A2P1 demonstrated the highest emulsion stability, followed by the A1P2. Hydrolyzed proteins reduce surface tension at the oil-water interface, acting as emulsifiers to facilitate emulsion formation through lipo-protein complexes with unique physicochemical properties.

At last, the antioxidant capacity of yeast proteins treated with complex enzymes is depicted in Fig. 6F. Proteins with strong antioxidant capacity can help prevent oxidative reactions in food products, by minimizing quality degradation, including undesirable flavors, textures, color changes, and reduction in nutritional value [16]. Also, hydrolytic reactions for peptide production are recognized as a primary method for generating protein-based antioxidants, given that peptides show significantly superior antioxidant potential compared to intact proteins [74]. Untreated yeast proteins demonstrated very low antioxidant activity, whereas enzymatically treated proteins displayed over 70% antioxidant capacity, regardless of enzyme ratio. The presence and arrangement of aromatic amino acids (tyrosine, tryptophan, histidine), sulfur-containing amino acids (cysteine, methionine), acidic amino acids (glutamic acid), and basic amino acids (lysine) are crucial for the functionality of antioxidant peptides [75]. However, amino acids with stable groups in their side chains, such as alkyl and phenyl groups, are inert towards oxidizing agents, exhibiting minimal or no antioxidative capability. This group includes valine, threonine, glycine, alanine, leucine, isoleucine, serine, aspartate, glutamine, glutamate, asparagine, phenylalanine, and proline [76]. Hydrophobic amino acid residues improve peptide solubility in lipid phases and facilitate interactions with free radicals, thereby enhancing their ability to inhibit lipid peroxidation [77]. This underscores the potential of yeast protein hydrolysates as antioxidants in food applications.

For the further researches, since the enzymatically hydrolyzed yeast protein showed potentiality as a functional alternative protein source, the investigation of applicability of the yeast protein across various food matrices or of bioactive properties for enhancing health benefits are recommended These could far enhance their potential as both nutritional and functional ingredients in health-focused alternative foods and nutraceuticals.

## Conclusion

In this study, the yeast proteins treated with a combination of endo- and exo-type enzymes at various complex ratios led to significant improvements in several key parameters. The yeast protein hydrolysates treated with A1P2 exhibited higher protein solubility and the highest WHC and OBC values. In the case of A2P1, it showed slightly larger particle sizes but more porous morphology and a significant enhancement in ESI. Additionally, enzymatical hydrolysis of yeast proteins resulted in elevated levels of EAAs and antioxidant activities, indicating improved protein quality. In conclusion, the combination of the endo- and exo-type proteases could give the functional and nutritional attributes of yeast proteins, positioning them as potential sustainable protein sources for a wide range of food applications.

## Figures and Tables

**Table 1:** Particle size of yeast protein by complex protease treatment.

A^a)^ to B^b)^ ratio	Diameter (μm)			Span
	D_10_	D_50_	D_90_	
1:1	3.78 ± 0.01^a^	9.76 ± 0.01^c^	20.20 ± 0.01^c^	1.68 ± 0.00^a^
1:2	3.74 ± 0.02^b^	10.04 ± 0.06^a^	20.60 ± 0.00^a^	1.68 ± 0.01^a^
2:1	3.74 ± 0.01^b^	9.92 ± 0.00^b^	20.40 ± 0.01^b^	1.68 ± 0.00^a^

^a)^Alcalase (A), ^b)^Prozyme 2000P (B)

Data are expressed as mean ± standard deviation (*n* = 3).

The means indicated with different letters within the same column are significantly different at *p* < 0.05.

**Table 2:** Gel permeation-chromatography data of yeast protein hydrolysate prepared with complex enzyme treatment.

A^a)^ to B^b)^ ratio	Mw	Mn	PDI^c)^
Yeast protein	6446.00 ± 50.00^c^	6438.50 ± 50.50^b^	1.00 ± 0.00^c^
1:1	8640.00 ± 30.00^b^	8136.50 ± 36.50^a^	1.06 ± 0.01^b^
1:2	8748.00 ± 50.00^a^	8222.00 ± 32.00^a^	1.07 ± 0.00^a^
2:1	8612.00 ± 12.00^b^	8134.50 ± 4.50^a^	1.06 ± 0.00^b^

^a)^Alcalase (A), ^b)^Prozyme 2000P (B), ^c)^Polydispersity index.

Data are expressed as mean ± standard deviation (*n* = 3).

The means indicated with different letters within the same column are significantly different at *p* < 0.05.

**Table 3:** Effect of enzyme treatment on SH content of yeast protein treated with complex enzyme.

A^a)^ to B^b)^ ratio	Total free SH group (μmol/g)	Surface SH group (μmol/g)
Yeast protein	4.54 ± 0.16^a^	2.55 ± 0.11^a^
1:1	1.72 ± 0.01^bc^	0.91 ± 0.03^cd^
1:2	1.74 ± 0.02^b^	0.73 ± 0.06^d^
2:1	1.55 ± 0.00^cd^	1.13 ± 0.01^c^

^a)^Alcalase (A), ^b)^Prozyme 2000P (B).

Data are expressed as mean ± standard deviation (*n* = 3).

The means indicated with different letters within the same column are significantly different at *p* < 0.05.

**Table 4:** Free amino acid composition of protein hydrolysates treated with various complex enzyme ratio.

Free amino acid	The ratio of enzyme-treatment A^a)^ to B^b)^		
	1:1	1:2	2:1
Aspartic acid	3195.26 ± 64.63^c^	3810.31 ± 78.17^b^	3419.97 ± 6.09^a^
Glutamic acid	4513.90 ± 115.92^b^	4932.50 ± 86.62^a^	4996.08 ± 42.38^a^
Asparagine	4576.11 ± 93.05^b^	5016.68 ± 95.72^a^	4448.36 ± 26.90^b^
Serine	4508.31 ± 80.89^b^	5608.7 ± 74.68^a^	4438.82 ± 42.12^b^
Glutamine	2569.49 ± 47.45^b^	3324.79 ± 52.70^a^	2679.92 ± 46.64^b^
Histidine	4528.62 ± 18.78^a^	4448.55 ± 139.84^a^	3833.72 ± 47.99^b^
Glycine	955.24 ± 17.65^c^	1411.76 ± 30.57^a^	1015.80 ± 10.18^b^
Threonine	8818.66 ± 139.46^a^	9060.01 ± 158.83^a^	7667.41 ± 88.89^b^
Citrulline	18.10 ± 0.27^a^	17.94 ± 1.87^a^	N.D.
Arginine	14480.10 ± 301.87^a^	17707.25 ± 308.13^a^	12654.83 ± 157.52^b^
Alanine	4496.46 ± 84.67^b^	5563.16 ± 97.73^a^	4210.14 ± 32.96^c^
Tyrosine	7325.96 ± 117.60^b^	7767.94 ± 79.52^a^	6258.32 ± 43.66^c^
Valine	10704.34 ± 243.61^b^	11250.35 ± 204.46^a^	9850.91 ± 76.01^c^
Methionine	3682.81 ± 72.85^b^	3850.35 ± 61.03^a^	3321.14 ± 32.76^c^
Tryptophane	1921.68 ± 30.91^b^	2069.18 ± 60.85^a^	1824.95 ± 26.02^b^
Phenylalanine	12226.88 ± 263.10^b^	12885.50 ± 229.41^a^	12369.91 ± 74.28^ab^
Isoleucine	8387.81 ± 184.12^b^	8864.68 ± 182.34^a^	7601.39 ± 73.03^c^
Leucine	18991.91 ± 347.75^b^	20036.29 ± 343.79^a^	18212.66 ± 248.70^b^
Lysine	12966.54 ± 251.62^b^	14184.41 ± 284.91^a^	12771.25 ± 251.20^b^
Proline	337.25 ± 44.17^a^	383.09 ± 2.59^a^	-
AAAs^c)^	21474.53 ± 402.61^b^	22722.61 ± 357.59^a^	20453.18 ± 142.49^c^
HAAs^d)^	63578.65 ± 128.13^b^	67107.37 ± 1143.66^a^	59439.29 ± 492.08^c^
EAAs^e)^	78546.45 ± 1362.66^b^	82798.95 ± 1595.66^a^	74132.21 ± 778.51^c^
TAA^f)^	129205.43 ± 2373.37^b^	139193.30 ± 2534.71^a^	121575.58 ± 1128.65^c^
HAAs/TAA (%)	49.21 ± 0.12^a^	48.21 ± 0.10^b^	48.89 ± 0.07^b^
EAAs/TAA (%)	60.79 ± 0.11^a^	59.48 ± 0.06^b^	60.98 ± 0.11^a^

^a)^Alcalase (A), ^b)^Prozyme 2000P (B)

^c)^AAAs: aromatic amino acids

^d)^HAAs: hydrophobic amino acids

^e)^EAAs: essential amino acids

^f)^TAAs: total amino acids

Data are expressed as mean ± standard deviation (*n* = 3).

The means indicated with different letters within the same column are significantly different at *p* < 0.05
